# AGE AS A DIVERSITY FACTOR IN HIGHER EDUCATION: INSIGHTS FROM DEI OFFICERS

**DOI:** 10.1093/geroni/igac059.1063

**Published:** 2022-12-20

**Authors:** Nancy Morrow-Howell, Natalie Galucia, Michele Dinman

**Affiliations:** Washington University in St. Louis, St. Louis, Missouri, United States; Washington University in St. Louis, St. Louis, Missouri, United States; Washington University in St. Louis, St. Louis, Missouri, United States

## Abstract

This study described how DEI officers across universities/colleges currently think about AGE as a diversity factor; and identified strategies used to increase age-inclusivity. Data were generated through review of university websites, focus groups and one-to-one interviews with DEI staff. Findings suggest that age is acknowledged as a diversity factor but there is less action toward strategies to increase age-inclusion. Examples of initiatives include: training human resource staff to be age-neutral in hiring; eliminating birthdates and other years from applications; workshops on multigenerational workplaces and classrooms; presentations on ageism; and specific programs to support non-traditionally aged students. Some of the motivation to address ageism stems from legal mandates rather than being mission-driven. There is the concern that focusing more on age may require moving attention and resources away from other diversity factors. It appears that there is interest in elevating age as an important factor in DEI efforts.

